# Exploring organizational readiness for the early adoption of a digital obesity prevention program in Swedish child healthcare: an observational prospective study

**DOI:** 10.1186/s12913-026-15953-6

**Published:** 2026-10-29

**Authors:** Maria Fagerström, Karin Festin, Maria Lundgren, Marie Löf, Jeanette Melin, Margit Neher, Christine Delisle Nyström, Kristin Thomas

**Affiliations:** 1 Department of Health, Medicine, and Caring Sciences, Linköping University, Linköping, Sweden; 2 Department of Medicine, Karolinska Institutet, Huddinge, Sweden; 3 Department of Health and Caring Sciences, Linnaeus University, Kalmar, Sweden; 4 Department of Leadership, Demand and Control, Swedish Defence University, Karlstad, Sweden; 5 Institute of Social Sciences, Södertörn University, Stockholm, Sweden; 6 School of Health and Welfare, Halmstad University, Halmstad, Sweden

**Keywords:** Organizational readiness, Child health services, Digital health, Adoption, Implementation science

## Abstract

**Background:**

Despite the emphasis on organizational readiness in implementation science, there is limited empirical data supporting its role in implementation practice. This study aimed to (a) assess dimensions of organizational readiness at implementation start of an evidence-based digital health promotion and obesity prevention program in Swedish child healthcare (MINISTOP) and (b) investigate the associations between assessed readiness dimensions and the adoption of MINISTOP.

**Methods:**

In this observational prospective study, all 135 child healthcare nurses from 48 Swedish child healthcare centers involved in the large-scale implementation of MINISTOP in 2023 were invited to participate. Organizational readiness, conceptualized as a multidimensional construct reflecting individual and collective aspects of readiness, was measured at baseline (*n* = 83 nurses from 37 centers) using the six-dimension E-Ready questionnaire. Adoption of MINISTOP was assessed at 19 weeks through registration data (i.e., the number of families who gained access to MINISTOP). Descriptive statistics and correlation analyses were employed to describe organizational readiness, while negative binomial regression was used to analyze associations between the E-Ready dimensions and adoption.

**Results:**

Nurses reported a high degree of readiness for the *conditions for change at the workplace*,* individual conditions for change* and *workplace attitudes* dimensions, a medium degree of readiness for the *perceived support among the management* dimension, and a low degree of readiness for the *readiness among colleagues* and *perceived consequences on status quo* dimension. No statistically significant associations were found between E-Ready’s dimensions and adoption. Nevertheless, positive estimates were observed for *readiness among colleagues* (IRR = 1.1, *p* = 0.05) and *conditions for change at the workplace* (IRR = 1.1, *p* = 0.08).

**Conclusion:**

No statistically significant associations were identified between organizational readiness and adoption. The findings illustrate the complexity of organizational readiness as a construct, while the positive estimates observed for collective aspects of readiness suggest that their potential relationship with adoption warrants further investigation. Longitudinal studies using repeated assessments are needed to clarify how organizational readiness relates to implementation outcomes over time.

**Trial registration:**

Not applicable.

**Supplementary Information:**

The online version contains supplementary material available at https://doi.org/10.1186/s12913-026-15953-6.

## Contributions to literature

- Although the concept of organizational readiness is repeatedly cited as an important determinant for implementation, empirical evidence supporting this premise is limited and varied, thus demonstrating the need for more research.
- These findings add to the knowledge base regarding the relationship between organizational readiness and implementation outcomes and highlight dimensions of organizational readiness that may warrant further investigation in relation to the adoption of digital interventions in Swedish child healthcare.
- The study makes a methodological contribution to implementation science by discussing and problematizing the concept of organizational readiness.

## Backgroud

Organizational readiness is a well-cited phenomenon within implementation science and has been proposed to explain implementation outcomes [1, 2]. Specifically, a healthcare organization’s success in implementing innovations or evidence-based practices is argued to rely on their organizational readiness, such as in terms of resources for change or management support [1, 2]. According to Weiner et al.’s theory [1], organizational readiness refers to the extent to which organizational members (e.g., staff, coworkers) are psychologically and behaviorally prepared to implement an upcoming change. In organizations with high readiness, members are more likely to take initiative, collaborate efficiently, work to overcome barriers, and implement to a greater extent, all of which ultimately results in higher implementation effectiveness [1]. In this manner, organizational readiness is considered a “shared team property” among members, although it can be assessed at the individual, group or organizational level [1]. Furthermore, key aspects of organizational readiness are incorporated into prevalent implementation science determinant frameworks such as the “Consolidated Framework for Implementation Research” (CFIR), in which organizational readiness has been categorized primarily within the inner setting domain where implementation occurs [3, 4]. While determinant frameworks, such as CFIR [3], help explain factors that may influence implementation, process models help describe how implementation unfolds over time [5]. One such model is the Exploration, Preparation, Implementation, Sustainment (EPIS) framework, which conceptualizes the implementation process across four phases: exploration, preparation, implementation, and sustainment [6]. From this process perspective, organizational readiness may be viewed as particularly relevant during the preparation phase and the transition to implementation, as organizations move from planning and preparing for a new intervention to actively integrating it into routine practice.

Despite the emphasis on organizational readiness in implementation science, there is limited empirical data supporting the theoretical claims of its role in implementation [2, 7]. Instead, much of the previous research has focused on assessing organizational readiness per se without analyzing readiness in relation to implementation outcomes [8–10]. The few existing studies that have explicitly examined the relationship between organizational readiness and implementation outcomes have investigated diverse clinical interventions across various healthcare settings [7, 11–13]. These studies have employed both quantitative [7, 11, 12] and mixed methods approaches [13], revealing somewhat varied results for different implementation outcomes. For example, Livet et al. [7], found that higher organizational readiness levels were associated with greater acceptability and feasibility of a medication management program in specialist healthcare, but not with perceived appropriateness. Bast et al. [11] observed that higher baseline organizational readiness correlated with higher fidelity to a smoking prevention program in schools. Regarding adoption, the findings are somewhat contradictory. Van Houtven et al. [12] noted that managers’ baseline perceptions of organizational readiness did not predict the adoption of a support program for caregivers in veteran medical organizations; however, organizations that adopted the program early reported higher baseline organizational readiness. Conversely, Peracca et al. [13] found a positive association between baseline organizational readiness and the adoption of a dermatology mobile app in Veteran Affairs facilities. In conclusion, summarizing the current knowledge on the importance of organizational readiness is challenging due to the limited and diverse nature of existing studies, which vary in results, contexts and interventions.

Digital health is a research area that emerged rapidly, particularly after the introduction of smartphones [14]. Consequently, a large number of digital interventions related to the prevention and treatment of various diseases have been developed to assist patients and healthcare providers [15]. Many digital interventions have shown evidence of effectiveness in promoting health at scale [16, 17]. One such intervention is MINISTOP, which has been researched for ten years via two randomized controlled trials and several qualitative studies [18–22]. MINISTOP is a digital obesity prevention program for families with preschool-aged children. It consists of the MINISTOP app for parents and the MINISTOP control tower for nurses (i.e., the child healthcare interface) [18]. Nurses use the interface to register the parents’ phone number so that they can download and access the app. The prevention program has been shown to have a significant effect on reducing the intake of sweets/sweetened drinks and the amount of screen time among children 2.5 to 3 years of age, as well as increasing parental self-efficacy for promoting healthy lifestyle behaviors [20]. In response to these positive findings, there has been great interest shown in implementing the program on a large scale within the Swedish child healthcare setting. Implementation of the current version of the app (MINISTOP 3.0, hereafter referred to as “MINISTOP”) in routine care was initiated in 2023. This development also provided us with a unique opportunity to conduct implementation research, so we designed a type 3 hybrid study with a published study protocol (the MINISTOP 3.0 trial) [18]. This trial includes several aims e.g., the evaluation of two implementation strategies (basic and enhanced) introduced after 20 weeks as well as clinical outcomes. In the current study, we focused on organizational readiness during the transition from preparation to early implementation of MINISTOP. To examine implementation under similar conditions across centers, we focused on the first 19 weeks following each center’s implementation start, before the implementation strategies differentiated the participating centers. Our aim was to (a) assess various dimensions of organizational readiness at the transition from preparation to implementation, and (b) investigate the associations between the assessed organizational readiness dimensions and the number of families gaining access to the app during the first 19 weeks of its implementation within the healthcare setting (i.e., measures of early adoption). Based on theoretical claims [1], we hypothesized that higher degrees of organizational readiness at implementation start would be associated with higher early adoption levels.

## Methods

### Study design

This study was designed as an observational prospective study. Questionnaire data was used to assess organizational readiness at baseline, as perceived by child healthcare nurses. Organizational readiness was conceptualized as a multidimensional construct reflecting both individual and collective aspects of readiness for implementation [23]. Readiness was operationalized using the E-Ready questionnaire [23], which assesses six dimensions related to readiness for implementing digital interventions in healthcare settings, including conditions for change at the workplace, individual conditions for change, perceived support and engagement among management, readiness among colleagues, perceived consequences on status quo, and workplace attitudes. Adoption was assessed as the individual child healthcare nurses’ total number of MINISTOP registrations in the child healthcare interface between baseline and the first 19 weeks of implementation.

### Research setting

This study was conducted within the Swedish child healthcare setting. The primary mission of Swedish child healthcare is to work to promote health and prevent disease for children 0 to 5 years of age. According to the Swedish national child healthcare program [24], children along with their parents are offered regular health visits at fixed intervals spanning from birth to five years of age. These health visits include monitoring growth and development, as well as conversations for promoting healthy lifestyle behaviors. This study focuses on routine health visits for children 2.5 to 3 years of age, hereafter referred to as “health visit (2.5-3 years)”.

Swedish child healthcare is organized as child healthcare centers, which can be both publicly and privately operated. Publicly operated centres are managed by regional authorities and financed through public taxation. Privately operated centres are managed by private healthcare providers but receive public funding through contracts with the regional healthcare system. Thus, both publicly and privately operated centres are financed through the publicly funded healthcare system. Child healthcare services are typically led by district or pediatric nurses. Their services are free of charge and reach 99% of all children in Sweden [25]. The child healthcare nurses that participated in this study were employed at various centers, all under the umbrella of the same healthcare company (privately run). During the study period, this healthcare company included 77 child healthcare centers.

#### The MINISTOP implementation process

The MINISTOP implementation process began when national-level decision-makers within a Swedish healthcare company formally decided to roll out MINISTOP across all 77 child healthcare centers. According to the EPIS framework [6], this decision and the subsequent planning activities can be viewed as part of the exploration and preparation phases. During the preparation phase, all child healthcare nurses were invited to a start-up educational meeting and received written educational materials regarding the use of MINISTOP [18].

This study included child healthcare centers that participated in these meetings from January 2023 until December 2023. The educational meetings were conducted at six timepoints during 2023, and centers could choose to attend the session most suitable for them. Centers attending the same educational meeting constituted an implementation start wave. Around the time for the meeting, nurses received access to the MINISTOP caregiver interface and could begin offering MINISTOP to families during routine child healthcare visits. Thus, in the present study, participation in the start-up educational meeting was conceptualized as marking the transition from preparation to implementation. Consequently, implementation was rolled out in six distinct start waves throughout the year. Table 1 displays the number of child healthcare centers and nurses involved in each start wave.

During 2023, MINISTOP was implemented in 48 out of the 77 child healthcare centers within the healthcare company. In the present study, the first 19 weeks following each center’s implementation start were conceptualized as an early implementation period. During this period, nurses were able to offer MINISTOP to families while centers were simultaneously establishing local routines and workflows related to the intervention.

The participating child healthcare centers varied in size: four child healthcare centers were small (1 employed child healthcare nurse), 39 were medium (2–4 employed child healthcare nurses), and five were large (more than 5 employed child healthcare nurses).

**Table 1:** Number of child healthcare centers and nurses included in the six different starting waves during 2023

Timepoint for implementation	Number of child healthcare centers (*n*)	Number of child healthcare nurses (*n*)
Wave 1	20	64
Wave 2	13	33
Wave 3	1	4
Wave 4	7	16
Wave 5	3	8
Wave 6	4	10

### Participants and recruitment

All 135 child healthcare nurses from the 48 centers that began the MINISTOP implementation in 2023 were invited via email to take part in the study. The inclusion criteria were (a) to currently work at a center implementing MINISTOP and (b) be able to provide the required data for the study, i.e., the total number of health visits conducted for children aged 2.5 to 3 years old during the study period. This data was vital to be able to analyze the degree of adoption. The exclusion criteria were (a) having worked at the center for less than three months, and (b) not remaining at the workplace throughout the entire study period.

### Measures

#### Organizational readiness

*Organizational readiness*, i.e., the independent variable, was measured with the E-Ready questionnaire [23] (Additional file 1). E-Ready was chosen based on its practical and comprehensible approach, which is well suited for use among healthcare professionals. Furthermore, the measure was developed in a Swedish healthcare context with a focus on digital interventions. The E-Ready questionnaire assesses perceived readiness for change at the individual and collective levels when implementing digital interventions within healthcare settings. The questionnaire consists of 29 items that are categorized into 6 dimensions: *conditions for change at the workplace* (8 items), *individual conditions for change* (3 items), *perceived support and engagement among management* (5 items), *readiness among colleagues* (5 items), *perceived consequences on status quo* (5 items), and *workplace attitudes* (3 items). In addition, E-Ready includes three single items assessing compatibility with current routines, individual commitment for change, and perceived need for change. Responses are given on a Likert scale, where wording and scale steps are tailored to each dimension. Five of the dimensions have scales ranging from 1 to 4, and one of the dimensions has a scale ranging from 1 to 5.

The E-Ready questionnaire was generated and evaluated through a formative evaluation process within Swedish healthcare settings via expert panels, cognitive interviews, and psychometrical tests using Rasch analyses. Further, the E-Ready questionnaire has been field-tested among Swedish healthcare professionals. Previous research shows that E-Ready exhibits structure and content validity as well as acceptable internal structure and fit validity. Within previous Rasch analyses, person reliability of the dimensions varied from low to moderate (0.56 to 0.87) [23].

Minor changes were made in the E-Ready questionnaire to tailor items to the child healthcare setting. This included using the term “introduce” instead of “implement” and specifying that the items concerned the child healthcare center’s organization. This specification was considered necessary since many child healthcare centers are interlinked with primary healthcare centers. The tailored version of the questionnaire was pilot tested among four child healthcare nurses. The nurses found the items to be clear and understandable, so no changes were made afterward.

#### Adoption

Adoption, i.e., the dependent variable, was operationalized as the child healthcare nurse’s delivery of MINISTOP to families during routine health visits (2.5-3 years). Adoption was measured at the individual level as the total number of registrations in the child healthcare interface between baseline and the first 19 weeks of implementation. One registration refers to that one family has accepted to get access to the MINISTOP app and that the nurse registered their phone number in the interface so that they can download and access the app.

#### Confounders

The confounders were selected based on empirical data [26] and consensus within the research group. Confounding variables included the total number of health visits (2.5-3 years) performed by each nurse during the study period (0–19 weeks), nurses’ years of experience in child healthcare, and the timepoint for MINISTOP’s implementation start (referred to as the “starting wave”). Starting wave was considered relevant because it was believed that the time of the year when implementation began could influence both readiness and adoption, such as if the implementation started just before holidays.

### Data collection

An email containing all the study information as well as a secure link to the informed consent form and baseline questionnaire was distributed to all nurses employed at participating child healthcare centers. Email addresses of eligible nurses were provided by the in-house project leader coordinating the MINISTOP implementation. The baseline questionnaire was distributed directly after the start-up educational meeting and included (1) the informed consent form, (2) the E-Ready questionnaire and (3) the sociodemographic questions (gender, age, number of years worked in child healthcare, and number of years worked at the current child healthcare center). Three reminders were sent via email within a period of ten days. After approximately seven days, the research team made a phone call to all the centers to address any queries regarding MINISTOP’s implementation and to remind them about the questionnaire.

Data on the number of health visits (2.5-3 years) conducted by each nurse during weeks 0 to 19 were collected via an administrative system that was linked to the child healthcare centers’ electronic health record system. Two child healthcare centers (with 3 responders each) lacked the administrative system, so nurses manually calculated the number of visits in the electronic health record system. Each child healthcare nurse’s delivery of MINISTOP to families during the health visits (2.5-3 years) during weeks 0 to 19 (adoption) was assessed through the number of registrations in the child healthcare interface.

### Statistical analysis

The E-Ready questionnaire dimensions were preprocessed using Rasch analyses to assess the alignment of observed ordinal data with the measurement model [27]. Analyses were conducted separately for each E-Ready dimension due to their weak correlations [23]. The three single items in the E-Ready were excluded from Rasch analyses. When the data fit the model, ordinal raw scores can be transformed into interval measures on a logit scale [27] representing each nurse’s degree of readiness across each E-Ready dimension [28].

For interpretability, these interval measures (hereafter “person measures”) were rescaled to a 0–10 scale while maintaining their linear properties [29]. For each E-Ready dimension, 0 represents the lowest degree of readiness and 10 the highest. As E-Ready lacks defined cut-off values, we classified the average of the transformed person measures as low (< 4), medium (between 4 and 6) and high (> 6).

Additional file 2 provides descriptive results from the single items and Additional file 3 presents the Rasch analyses.

Descriptive statistics were used to describe and summarize the demographic data and to describe the degrees of readiness across each E-Ready dimension as well as to present the three single items from the E-Ready questionnaire. The Pearson correlation coefficient was used to investigate correlations between the person measures of the E-Ready dimensions as well as to investigate correlations between the demographic factors and person measures of the E-Ready dimensions. The Spearman correlation coefficient was used to investigate correlations between the demographic factors and the frequency of registrations in the child healthcare interface (adoption).

A one-way ANOVA was conducted to compare the starting waves based on the centers’ size, nurses’ total experience in child healthcare, and adoption levels. Dunnett’s T3 post hoc test was used for the subsequent comparisons due to unequal group sizes and variances.

Negative binomial regression models were used to investigate the associations between organizational readiness (per dimension of E-Ready) and the number of registrations in the child healthcare interface during weeks 0–19, and to investigate the potential influence of confounders. This approach was chosen because of the count nature of the dependent variable and its overdispersion. All nurses conducted at least four eligible health visits during the study period. Thus, within the relatively short 19-week early implementation period, zero registrations were considered more likely to reflect that adoption had not yet occurred than a complete absence of opportunity to offer the intervention. The analyses were conducted in three steps. Model 1 was unadjusted. Model 2 was adjusted for each nurse’s total number of conducted health visits (2.5-3 years) during weeks 0 to 19 and number of years of experience in child healthcare. Model 3 included an additional adjustment for the implementation timepoint (i.e., starting wave) to assess whether a starting wave was associated with the outcomes. Starting wave five had only one respondent, so starting waves five and six were merged together for this analysis. For this analysis, we hypothesized that starting later (waves 5/6) would be associated with better readiness, so this was fitted as the reference category. The individual nurse was the unit of analysis, as both readiness and adoption were measured at the individual level. Nurses typically worked independently with the families assigned to them and made individual decisions about offering MINISTOP, even when several nurses were employed at the same center. The regression models did not account for potential clustering within child healthcare centers. A p-value of < 0.05 was considered statistically significant. Regression assumptions were verified, including linearity and multicollinearity checks. Additionally, model fit was confirmed through residual analyses.

For the Rasch analyses, data were analyzed according to the unrestricted polytomous Rasch model using RUMM 2030 (version 5.8.1). For descriptive and regression statistics, we used IBM SPSS Statistics version 29. We followed the STROBE checklist for study reporting (Additional file 4).

## Results

### Descriptive results

All nurses working at centers implementing MINISTOP (*n* = 135 nurses from 48 child healthcare centers) were invited to take part in the study. Of these, 89 nurses from 40 different child healthcare centers provided their informed consent and completed the baseline questionnaire with no missing data. A total of five participants did not meet the inclusion criteria since they could not provide the required administrative data on the total number of routine health visits (2.5-3 years) during the study period (confounding factor). One participant was excluded due to taking parental leave within the 19-week study period. As a result, data from 83 participants from 37 different child healthcare centers were analyzed. Of these, 41 were from the first wave, 23 from the second wave, two from the third wave, 12 from fourth wave, one from the fifth wave, and four from the sixth wave. The six different starting waves did not differ in terms of the nurses’ age (*p* = 0.09), or their total experience in child healthcare (*p* = 0.4). However, a significant difference was detected between the waves in terms of size, i.e., the number of nurses employed at the centers (*p* = 0.04). Specifically, the first wave included larger centers compared to the second wave (mean difference = 1.6, *p* = 0.05) and fourth wave (mean difference = 2.0, *p* = 0.02), and the third wave included larger centers compared to the second wave (mean difference = 0.7, *p* = 0.02). Correlation analyses showed that the later starting waves included fewer employed nurses (*r* = − 0.3, *p* = 0.01).

**Fig. 1:**
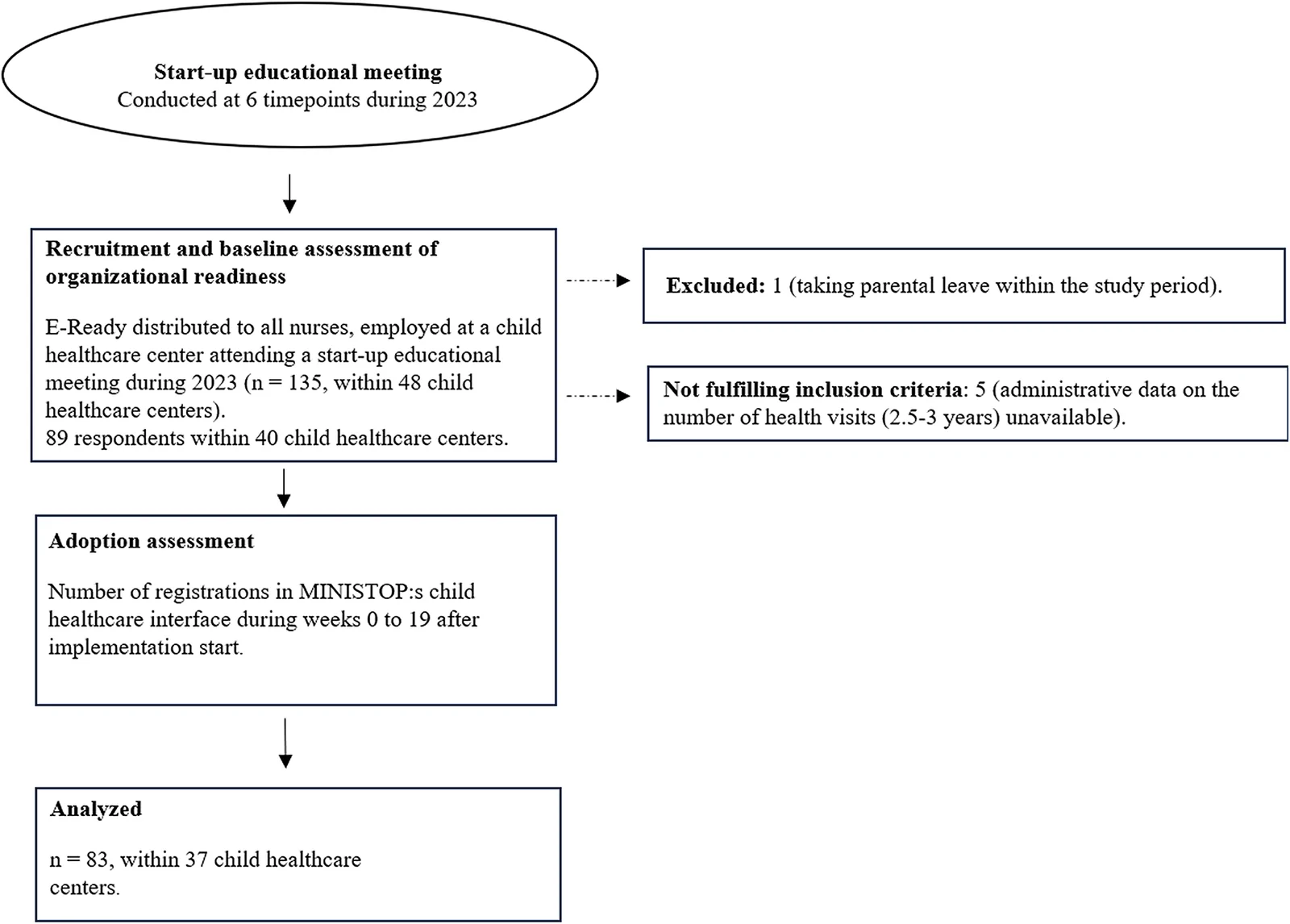
Summary of the study process

Figure 1 summarizes the study process. Descriptive characteristics of the study participants are shown in Table 2.

**Table 2:** Participant and organizational characteristics

Participant characteristics (*n* = 83)	
*Age of nurse*	
Frequency, n (%)	
30 years or under	0 (0)
31–40 years	31 (37)
41–50 years	29 (35)
51 years or above	23 (28)
Mean (SD)*	45 (9)
Median (IQR)*	43 (16)
*Gender* Frequency, n (%)	
Male	0 (0)
Female	82 (99)
Preferred not to disclose	1 (1)
*Years of experience in child healthcare* Frequency, n (%)	
≤ 3	15 (18)
3–6	28 (34)
7–10	16 (19)
≥ 10	24 (29)
Mean (SD)*	8 (7)
Median (IQR)*	6 (9)
*Years of experience at current workplace* Frequency, n (%)	
≤ 3	19 (23)
3–6	37 (45)
7–10	16 (19)
≥ 10	11 (13)
Mean (SD)*	6 (4)
Median (IQR)*	5 (4)
*Total number of health visits (2.5-3 years) conducted per nurse within the first 19 weeks of implementation*	
Range, min; max	4;57
Mean (SD)*	25 (10)
Median (IQR)*	24 (12)
**Organizational characteristics (n = 37)**	
*Clinic size (number of employed nurses)* Frequency n (%)	
1	3 (8)
2–4	29 (78)
≥ 5	5 (14)
Mean (SD)* Median (IQR)*	3 (2) 3 (2)

*SD = Standard Deviation

*IQR = Interquartile Range

### Degrees of readiness

Table 3 presents the means and medians of the transformed person measures, as well as the classification of readiness degrees across the E-Ready dimensions. Although *perceived support among the management* showed a medium degree of readiness, it also displayed a large standard deviation, indicating variability among the nurses’ perceived readiness.

**Table 3:** Means, medians of transformed person measures and readiness degree classifications for each E-Ready dimension (*n* = 83)

E-Ready dimension	Mean person measure* (SD)*	Degree of readiness*	Median person measure* (IQR)*
Conditions for change at the workplace	6.2 (1.5)	High	6.0 (1.5)
Individual conditions for change	7.7 (1.7)	High	7.6 (2.4)
Perceived support and engagement among management	4.6 (3.1)	Medium	4.6 (3.9)
Readiness among colleagues	3.9 (2.0)	Low	4.3 (1.8)
Perceived consequences on status quo	3.5 (2.1)	Low	3.4 (2.5)
Workplace attitudes	7.7 (1.8)	High	8.1 (2.3)

*A person measure of 0 represents the lowest degree of readiness and 10 the highest

*SD = Standard Deviation

*Degrees of readiness: mean below 4 = low, 4–6 = medium, and above 6 = high

*IQR = Interquartile Range

Correlation analyses indicated that working at larger centers was associated with higher degrees for *perceived support and engagement among management* (*r* = 0.3, *p* = 0.004). Furthermore, more work experience within child healthcare was associated with lower degrees for *perceived consequences on status quo* (*r* = − 0.2, *p* = 0.04). Additionally, later starting waves were associated with lower degrees of *workplace attitudes* (*r* = -0.3, *p* = 0.001). No other statistically significant correlations were found between the degrees of readiness for other E-Ready dimensions and sociodemographic data or confounding variables, i.e., the nurses’ age, current workplace experience, total work experience in child healthcare, number of employed nurses at the child healthcare center or starting wave.

The person measures across the E-Ready dimensions generally correlated with each other (p-values ranging from < 0.001 to 0.02), except for *“individual conditions for change”* which did not correlate with *perceived support and engagement with the management* (*p* = 0.5) or *perceived consequences on status quo* (*p* = 0.15) (see Table 4). However, all the correlations ranged from very weak to moderate [30], indicating that high levels in one dimension did not necessarily correspond with high levels in another.

**Table 4:** Correlations among person measures across the E-Ready dimensions

	Conditions for change at the workplace	Individual conditions for change	Perceived support and engagement among management	Readiness among colleagues	Perceived consequences on status quo	Workplace attitudes
Conditions for change at the workplace	1					
Individual conditions for change	0.4 (*p* < 0.001)	1				
Perceived support and engagement among management	0.3 (*p* = 0.009)	0.08 (*p* = 0.5)	1			
Readiness among colleagues	0.5 (*p* < 0.001)	0.3 (*p* = 0.02)	0.3 (*p* = 0.003)	1		
Perceived consequences on status quo	0.5 (*p* < 0.001)	0.2 (*p* = 0.15)	0.3 (*p* = 0.02)	0.3 (*p* = 0.007)	1	
Workplace attitudes	0.4 (*p* < 0.001)	0.4 (*p* < 0.001)	0.2 (*p* = 0.03)	0.3 (*p* = 0.001)	0.5 (*p* < 0.001)	1

### Adoption levels

During weeks 0–19, the total number of registrations per nurse in the child healthcare interface ranged from 0 to 29, with data being highly skewed and clustered at zero. The median number of registrations was 2 (IQR 6). In total, 34 nurses (41%) did not make any registrations during these weeks, while 49 (59%) made at least one registration. There were no statistically significant correlations between the nurses’ age, current workplace experience, total work experience in child healthcare, starting wave, number of employed nurses at the child healthcare center, total number of health visits (2.5-3 years) and the number of registrations in the MINISTOP child healthcare interface. Additionally, adoption levels did not significantly differ between the different starting waves.

### Association between organizational readiness and adoption

Table 5 shows the result of the negative binomial regression models. All Pearson Chi-Square model fit tests indicated an adequate fit of the data to the model, with values ranging from 1.3 to 1.4.

Positive estimates were observed for *readiness among colleagues* and *conditions for change at the workplace.* For each one-unit increase in the logit of *readiness among colleagues*, the estimated number of registrations increased by a factor of 1.1 (IRR = 1.1 95% CI = 1.0-1.2, *p* = 0.05). A similar estimate was observed for *conditions for change at the workplace* (IRR = 1.1, 95% CI 1.0-1.3, *p* = 0.08). However, neither association was statistically significant.

No statistically significant associations with adoption were found for *individual conditions for change*, *perceived support and engagement among management*, *perceived consequences on status quo* or *workplace attitudes*.

We found that certain confounders were of significance for the number of MINISTOP registrations. Regardless of the dimension, a higher number of health visits (2.5-3 years) conducted during weeks 0 to 19 was associated with an increased number of registrations during the same period (IRR = 1.04 for all dimensions, p-values ranging from 0.01 to 0.02). Additionally, differences between the starting waves were observed across the models. Wave four had higher estimated adoption than waves 5/6, with IRRs ranging from 10.8 to 12.6 (p-values between 0.001 and 0.003). However, these estimates should be interpreted with caution due to the small number of nurses in some starting waves and the wide confidence intervals.

No statistically significant association was found between the nurses’ experience in child healthcare and the number of registrations in the child healthcare interface.

**Table 5:** Negative binomial regression between E-ready dimensions and total number of MINISTOP registrations during weeks 0–19

Variable	MODEL 1*		MODEL 2*		MODEL 3*	
	IRR* (95% CI)*	*p*-value	IRR* (95% CI)*	*p*-value	IRR* (95% CI)*	*p*-value
**Conditions for change at the workplace**	1.2 (1.0;1.3)	0.03	1.2 (1.0;1.3)	0.02	1.1 (1.0;1.3)	0.08
Number of health visits			1.0 (1.0;1.1)	0.03	1.0 (1.0;1.1)	0.01
Years of experience in child healthcare			1.0 (0.9;1.0)	0.13	1.0 (0.9;1.0)	0.20
Timepoint for implementation						
*Wave 1*					4.2 (0.9;18.8)	0.06
*Wave 2*					4.4 (0.9;21.6)	0.07
*Wave 3*					2.4 (0.2;24.9)	0.50
*Wave 4*					10.8(2.2;52.7)	0.003
*Waves 5 and 6 (reference group)*					-	
**Individual conditions for change**	1.0 (0.9;1.1)	0.82	1.0 (0.9;1.1)	0.97	1.0 (0.9;1.1)	0.95
Number of health visits			1.0 (1.0;1.1)	0.04	1.0 (1.0;1.1)	0.02
Years of experience in child healthcare.			1.0 (0.9;1.0)	0.08	1.0 (0.9;1.0)	0.14
Timepoint for implementation						
*Wave 1*					4.5 (1.1;18.3)	0.04
*Wave 2*					4.0 (0.9;18.0)	0.07
*Wave 3*					2.2 (0.2;21.7)	0.49
*Wave 4*					11.5 (2.6;51.2)	0.001
*Waves 5 and 6 (reference group)*					-	
**Perceived support and engagement among management**	1.1 (1.0;1.1)	0.21	1.1 (1.0;1.2)	0.19	1.0 (0.9;1.1)	0.94
Number of health visits			1.0 (1.0;1.1)	0.04	1.0 (1.0;1.1)	0.02
Years of experience in child healthcare			1.0 (0.9;1.0)	0.08	1.0 (0.9;1.0)	0.14
Timepoint for implementation						
*Wave 1*					4.5 (1.1;18.1)	0.04
*Wave 2*					4.1 (0.9;18.2)	0.07
*Wave 3*					2.2 (0.2;21.9)	0.49
*Wave 4*					11.4 (2.5;51.4)	0.002
*Waves 5 and 6 (reference group)*					-	
**Readiness among colleagues**	1.1 (1.0;1.1)	0.09	1.1 (1.0;1.2)	0.04	1.1 (1.0;1.2)	0.05
Number of health visits			1.0 (1.0;1.1)	0.03	1.0 (1.0;1.1)	0.01
Years of experience in child healthcare			1.0 (0.9;1.0)	0.05	1.0 (0.9; 1.0)	0.09
Timepoint for implementation						
*Wave 1*					4.8 (1.1;21.1)	0.04
*Wave 2*					4.8 (1.0;23.4)	0.05
*Wave 3*					2.2 (0.2;22.3)	0.51
*Wave 4*					12.6 (2.6;60.6)	0.002
*Waves 5 and 6 (reference group)*					-	
**Perceived consequences on status quo**	1.0 (0.9;1.2)	0.58	1.0 (0.9;1.2)	0.74	1.0 (0.9;1.1)	0.79
Number of health visits			1.0 (1.0;1.1)	0.04	1.0 (1.0;1.1)	0.02
Years of experience in child healthcare			1.0 (0.9;1.0)	0.10	1.0 (0.9;1.0)	0.13
Timepoint for implementation						
*Wave 1*					4.6 (1.1;18.7)	0.04
*Wave 2*					4.1 (0.9;18.5)	0.06
*Wave 3*					2.2 (0.2;21.7)	0.49
*Wave 4*					11.8 (2.6;53.3)	0.001
*Waves 5 and 6 (reference group)*					-	
**Workplace attitudes**	1.0 (0.9;1.1)	0.50	1.0 (0.9;1.1)	0.46	1.0 (0.9;1.2)	0.72
Number of health visits			1.0 (1.0;1.1)	0.04	1.0 (1.0;1.1)	0.02
Years of experience in child healthcare			1.0 (0.9;1.0)	0.07	1.0 (0.9;1.0)	0.14
Timepoint for implementation						
*Wave 1*					4.2 (1.0;17.7)	0.05
*Wave 2*					4.0 (0.9;18.0)	0.07
*Wave 3*					2.2 (0.2;21.5)	0.50
*Wave 4*					11.1 (2.50;49.9)	0.002
*Waves 5 and 6 (reference group)*					-	

*Model 1 was unadjusted. Model 2 was adjusted for each nurse’s total number of conducted health visits (2.5-3 years) during weeks 0 to 19 and their years of experience in child healthcare. Model 3 included an additional adjustment for the timepoint for implementation (starting wave)

*IRR = Incidence Rate Ratio

*CI = Confidence Interval; mean plus/minus 1.96 standard errors

## Discussion

Assessing organizational readiness before implementation can provide valuable insights into potential areas within a workplace that need to be improved or managed for successful implementation. However, empirical data confirming a link between organizational readiness and implementation outcomes is limited [2, 7, 31]. This study aimed to fill part of this knowledge gap by assessing various dimensions of organizational readiness at the transition from preparation to implementation of a digital obesity prevention program (MINISTOP) in Swedish child healthcare, and to investigate the associations between the assessed organizational readiness dimensions and the number of families gaining access to MINISTOP during the first 19 weeks of implementation (i.e., adoption).

We posited that a higher degree of organizational readiness at implementation start would be associated with higher subsequent adoption levels within the first 19 weeks of implementation. This assumption was not statistically confirmed. However, although no statistically significant associations were identified, positive estimates were observed for *readiness among colleagues* and *conditions for change at the workplace*, with adoption. Nevertheless, these findings should be interpreted with caution and considered hypothesis-generating, warranting further investigation. We also found that nurses reported varying degrees of organizational readiness across the E-Ready dimensions. Specifically, high degrees of organizational readiness were reported for *conditions for change at the workplace*,* individual conditions for change*, and *workplace attitudes*, medium degrees for *perceived support among the management*, and low degrees for *readiness among colleagues* and *perceived consequences on status quo*.

The E-Ready *readiness among colleagues* dimension involves discussing implementation with coworkers and sharing responsibility. The importance of this aspect for readiness aligns with findings from our previous qualitative study on organizational readiness to implement MINISTOP in child healthcare, where we found that trustful relationships among colleagues were perceived to facilitate new routines and enhance collaborative learning about the app [32]. Combined, findings from both these studies suggest that the relational aspects of organizational readiness and adoption warrant further investigation.

Our results complement previous research on the relationship between organizational readiness and implementation outcomes. Livet et al. [7] found that baseline organizational readiness was associated with early implementation outcomes, such as the acceptability, feasibility and intent to adopt a medication management program. However, these early implementation outcomes can be seen as *attitudes* towards an upcoming change, i.e., potential determinants for adoption [33]. In contrast, Van Houtven et al. [12] found no significant relationship between organizational readiness and the adoption of a support program for caregivers of veterans within one year. However, among sites that adopted the program, higher organizational readiness was associated with earlier adoption. As discussed by Van Houtven et al. [12], and consistent with theory, readiness is dynamic and changes throughout the implementation process [2]. Although not statistically confirmed, the positive estimates observed in our study, together with Van Houtven et al.’s [12] findings regarding earlier adoption, highlight the need for further research examining the relationship between organizational readiness and adoption at different stages of implementation.

In further consideration of Van Houtven et al.’s results [12], there is a risk that the nurses in our study might have adopted MINISTOP at later stages than our measurement timepoint. Determining the appropriate timepoint to measure adoption can be challenging, at times even arbitrary, and vary between contexts and clinical interventions. In our study, 19 weeks was the maximum possible period to study adoption due to interference with related research projects [18]. However, 19 weeks might have been too early since many child healthcare centers only recently transitioned from preparation to implementation and were still establishing routines related to the MINISTOP. Future research should measure readiness and adoption repeatedly and over a longer period of time.

Furthermore, the results suggested that estimated adoption levels may have differed across the different starting waves, with higher estimates observed for waves one and four compared with waves 5/6. However, the small and highly unequal group sizes, particularly in waves three and five, resulted in wide confidence intervals and unstable estimates. These findings should therefore be interpreted with caution and should not be regarded as evidence of systematic differences in adoption between the starting waves. Descriptively, the waves did not differ with regard to nurses’ ages or clinical work experience. Although some differences in center sizes were identified, center size was not associated with adoption levels. The reasons for the observed variation therefore remain unclear. Given the complexity of large-scale implementation across multiple child healthcare centers, it is unlikely that all contextual influences could be captured within the present study. Future research is needed to better understand how contextual and organizational factors may influence adoption during early implementation.

We found that the different readiness dimensions only had very weak to moderate correlation with each other. This prompts us to consider whether the concept of organizational readiness can be viewed as a single phenomenon. The question of what organizational readiness encompasses is not new, and the theoretical concept presents several challenges [4, 31]. In 2020, Weiner et al. [31] identified 11 different definitions of organizational readiness that have been proposed since Armenakis’ initial definition in 1993 [34]. These definitions vary both in what the construct encompasses, such as psychological, affective and/or structural components, as well as their level of analysis [31]. Literature also uses different words for organizational readiness, such as preparedness, willingness, barriers/facilitators or commitment [35]. Findings from a recent study by Annerstedt et al. [36] showed that early implementation outcomes (acceptability, appropriateness and feasibility), when measured simultaneously with organizational readiness, are highly correlated with each other. Indeed, this raises the question of whether these proxy outcomes and organizational readiness even cover similar phenomena.

Our results showed that nurses exhibited low readiness concerning *perceived consequences on status quo*. This E-Ready dimension involves how MINISTOP affects the nurses’ daily work, including its influence on their ability to manage their tasks and fulfill their professional responsibilities. This finding aligns with previous research which suggests that child healthcare nurses may view health-promoting digital tools as conflicting with their professional role [32]. In line with the CFIR framework [3], the compatibility of an innovation with existing workflows, routines, and values may act as either a barrier or a facilitator to implementation. In the present study, nurses’ perceptions regarding how MINISTOP affected their daily work and professional responsibilities may reflect such compatibility-related determinants. Additionally, our findings show that the nurses experienced lower *readiness among colleagues* and perceived a variation in *management support and engagement.* This issue may be attributed to the organizational structure of child healthcare centers in Sweden. Typically, nurses work independently, often with few or no colleagues in the same profession, which can limit opportunities for valuable peer discussions. Furthermore, child healthcare centers are affiliated with primary healthcare centers, wherein the smaller centers share the same manager as the primary healthcare centers, while the larger child healthcare centers have dedicated managers. Insights from our previous qualitative study revealed that nurses perceive primary healthcare management as lacking awareness of child healthcare center-specific issues [32]. Instead, changes are largely driven by national child healthcare service goals and governance [32]. Our findings also showed that nurses working at larger centers reported higher perceived leadership engagement and support and that smaller centers enrolled in the implementation later. Nevertheless, leadership engagement is crucial for effective implementation [3]. Addressing leadership issues in child healthcare centers could enhance future implementation efforts.

### Methodological discussion

This study is strengthened by being conducted across multiple child healthcare centers. Additionally, the validity of the findings was enhanced by involving all the child healthcare center nurses in the process of assessing the study parameters rather than relying on responses from a single representative [31]. Indeed, employees can experience organizational readiness differently, suggesting that answers from a broader spectrum of employees may provide a more reliable picture of readiness [31]. The validity is further supported by measuring adoption objectively through the child healthcare interface, which provides the exact number of MINISTOP registrations.

Nurses’ low level of MINISTOP registrations during this early implementation period may have limited our ability to statistically identify significant associations between adoption and organizational readiness. Future research, assessing adoption at later stages of implementation, when organizations have had more time to integrate an intervention into routine practice, could yield deeper insights into the relevance of organizational readiness for adoption. Assessing adoption at later stages was not feasible in the present study. This study only included centers that enrolled the first year of the MINISTOP implementation. These centers may exhibit higher readiness and adoption levels compared to those implementing it later. Additionally, the centers could choose from six different starting points for implementation based on what suited them best. Since readiness naturally changes over time [2], centers likely chose to start when they felt most ready. A simultaneous start for all centers would potentially have provided a wider range of readiness levels but was not feasible due to logistics of the large-scale implementation. Furthermore, child healthcare nurses who responded to the questionnaire may have considered themselves readier than those who did not respond. Finally, although nurses typically worked independently, those employed at the same centre may have shared organizational conditions. The regression models did not account for potential within-centre correlation, which may have affected the precision of the estimates and should be considered when interpreting the findings.

One strength of this study was its use of linear data to measure organizational readiness, made possible by the Rasch analyses. Linear data offers advantages over analyzing ordinal raw scores and provides a more precise measurement of a construct. Our results should also be interpreted in light of these psychometric analyses (see Additional file 3). E-Ready was developed relatively recently, and its main drawback was that some questions could potentially predict the answers of other questions [28]. Once we addressed this issue in our psychometric analyses, however, the ordinal data fit relatively well into the mathematical model. Nevertheless, two dimensions (*individual conditions for change* and *workplace attitudes*) exhibited ceiling effects and lower reliability (PSI 0.68 and 0.59). This was attributed primarily to the fact that these dimensions only contain three items. With such a small number of items, there is limited information available for estimating person measures.

## Conclusion

Overall, the nurses reported varying degrees of organizational readiness across the E-Ready dimensions during the implementation of a digital obesity prevention program in Swedish child healthcare. Although no statistically significant associations were identified between organizational readiness and nurses’ adoption of the intervention, the positive estimates observed for collective aspects of readiness may inform hypotheses for future research on implementation outcomes. Further research is needed to clarify how organizational readiness should best be conceptualized and to examine associations between readiness and implementation outcomes more closely. We suggest that future research explore this relationship over a longer period of time using repeated assessments of both readiness and implementation outcomes.

## Supplementary Information

Below is the link to the electronic supplementary material.

Supplementary Material 1

Supplementary Material 2

Supplementary Material 3

Supplementary Material 4

## Data Availability

Data that supports the research findings are available from the corresponding author upon reasonable request.

## Abbreviations

CFIR: Consolidated Framework for Implementation Research
EPIS: Exploration, Preparation, Implementation, Sustainment framework
