# Supplementary material for: Exploring organizational readiness for the early adoption of a digital obesity prevention program in Swedish child healthcare: an observational prospective study
Source: BMC Health Serv Res. 2026 Oct 29;26:1483. doi: 10.1186/s12913-026-15953-6 (PMC13721193; doi:10.1186/s12913-026-15953-6)
Supplement: Supplementary file 1 [file 12913_2026_15953_MOESM1_ESM.pdf]

Additional file 1.
The E-Ready questionnaire

# E-Ready©[1] Questionnaire

1. How do you perceive the **resources in your workplace** to implement X?

| | Does not exist at all | Exist to a small extent | Exist to a fairly large extent | Exist to a large extent |
|---|---|---|---|---|
| a) Sufficient allocated time to implement X | ☐ | ☐ | ☐ | ☐ |
| b) Sufficient staff resources to implement X | ☐ | ☐ | ☐ | ☐ |
| c) Clear goals on how to implement X | ☐ | ☐ | ☐ | ☐ |
| d) Experience among colleges to work with digital solutions | ☐ | ☐ | ☐ | ☐ |
| e) Experience among colleges to implement new work routines | ☐ | ☐ | ☐ | ☐ |
| f) Adequate competency to implement X | ☐ | ☐ | ☐ | ☐ |
| g) Sufficient support and guidance to adapt X to our workplace | ☐ | ☐ | ☐ | ☐ |
| h) Sufficient training to be able to use and work with X | ☐ | ☐ | ☐ | ☐ |

Comments:

___________________________________________________________________________

____________________________________________________________________

[1] Although copyrighted, E-Ready may be freely used if appropriate credit is given. Please contact Dr. Petra Dannapfel (petra.dannapfel@sll.se) if you intend to use E-Ready. Please note that the English translation presented here is for the purpose of this paper only. Translations of E-Ready from Swedish to several languages is currently under way.

Additional file 1.
The E-Ready questionnaire

2. How do you perceive **your own resources** to use X in your work?

| | Does not exist at all | Exist to a small extent | Exist to a fairly large extent | Exist to a large extent |
|---|---|---|---|---|
| a) Experience to work with digital solutions | ☐ | ☐ | ☐ | ☐ |
| b) Competency to work with X | ☐ | ☐ | ☐ | ☐ |
| c) Feeling comfortable to work with X | ☐ | ☐ | ☐ | ☐ |

Comments:

_______________________________________________________________________________

3.To what extent do you perceive your **manager** ...

| | Not at all | To some extent | Fairly large extent | Large extent |
|---|---|---|---|---|
| a) Communicates the need for implementing X | ☐ | ☐ | ☐ | ☐ |
| b) Encourages staff to engage in activities to implement X | ☐ | ☐ | ☐ | ☐ |
| c) Takes an active role in implementing X | ☐ | ☐ | ☐ | ☐ |
| d) Clearly communicates with staff how X will be implemented | ☐ | ☐ | ☐ | ☐ |
| e) Have insights in how X will influence status quo | ☐ | ☐ | ☐ | ☐ |

Comments:

_______________________________________________________________________________

4. To what extent do you perceive your **colleagues**...

| | Not at all | To some extent | Fairly large extent | Large extent |
|---|---|---|---|---|
| a) Discuss how work routines need to change when implementing X | ☐ | ☐ | ☐ | ☐ |
| b) Discuss duties that need to be omitted when implementing X | ☐ | ☐ | ☐ | ☐ |
| c) Discuss new duties that needs to be done when implementing X | ☐ | ☐ | ☐ | ☐ |
| d) Work together to adapt current work routines to X | ☐ | ☐ | ☐ | ☐ |
| e) Take collective responsibility for the implementation | ☐ | ☐ | ☐ | ☐ |

Comments:

___

5. In what way do you think implementing X will **impact your daily routines** regarding...

| | Considerably worse | Somewhat worse | Unchanged | Slightly better | Much better |
|---|---|---|---|---|---|
| a) Your ability to work in accordance with your values | ☐ | ☐ | ☐ | ☐ | ☐ |
| b) Your ability to exercise your professional role | ☐ | ☐ | ☐ | ☐ | ☐ |
| c) Your ability to offer high quality care | ☐ | ☐ | ☐ | ☐ | ☐ |
| d) Your ability to manage all your work | ☐ | ☐ | ☐ | ☐ | ☐ |
| c) How you think X aligns with the current workflow at your workplace? | ☐ | ☐ | ☐ | ☐ | ☐ |

Comments:

______________________________________________________________________

6. How do you perceive attitudes to implement X at **your workplace?**

| | Very negative | Rather negative | Rather positive | Highly positive |
|---|---|---|---|---|
| a) Among colleagues | ☐ | ☐ | ☐ | ☐ |
| b) Your attitude | ☐ | ☐ | ☐ | ☐ |
| c) Your manager’s attitude | ☐ | ☐ | ☐ | ☐ |

Comments:

______________________________________________________________________

| | Not at all | Quite bad | Quite good | Very good |
|---|---|---|---|---|
| 7. How well does X fit with current work routines? | ☐ | ☐ | ☐ | ☐ |

Comments:

______________________________________________

| | Passive | Rather passive | Rather active | Active |
|---|---|---|---|---|
| 8. What role would you like to have when X is being implemented at your workplace? | ☐ | ☐ | ☐ | ☐ |

Comments:

______________________________________________

| | Pointless | Some value | Fairly great value | Great value |
|---|---|---|---|---|
| 9. How do you perceive the value of implementing X at your workplace? | ☐ | ☐ | ☐ | ☐ |

Comments:

______________________________________________
