# Supplementary material for: Exploring organizational readiness for the early adoption of a digital obesity prevention program in Swedish child healthcare: an observational prospective study
Source: BMC Health Serv Res. 2026 Oct 29;26:1483. doi: 10.1186/s12913-026-15953-6 (PMC13721193; doi:10.1186/s12913-026-15953-6)
Supplement: Supplementary file 2 [file 12913_2026_15953_MOESM2_ESM.pdf]

Descriptive results: Single items of E-Ready

The E-Ready questionnaire includes three single items that were not included in the Rasch analyses and the main results of the study. This section provides a descriptive summary of these items showing the numbers and percentages of respondents who selected each response category.

Single item 1

For *compatibility with current work routines*, 12 nurses (14%) considered MINISTOP to have a very good fit. A total of 63 nurses (76%) stated quite a good fit. 8 nurses (10 %) reported that MINSTOP had quite poor compatibility, while no one (0%) reported that it didn't fit at all.

Single item 2

In terms of *commitment for change*, 26 nurses (31%) reported that they intended to take an active role in the implementation of MINISTOP, while 53 nurses (64%) stated that they intended to take quite an active role. 4 persons (6%) aimed to take a quite passive role and (0%) a passive role.

Single item 3

As for the *perceived advantage* of implementing MINISTOP, 26 persons (31%) considered the implementation of MINISTOP to be highly advantageous. 46 nurses (55%) saw it as being quite advantageous, and 11 nurses (13%) perceived it as being only slightly advantageous. No one (0 %) reported that it offered no advantage at all.
