# Supplementary material for: Exploring organizational readiness for the early adoption of a digital obesity prevention program in Swedish child healthcare: an observational prospective study
Source: BMC Health Serv Res. 2026 Oct 29;26:1483. doi: 10.1186/s12913-026-15953-6 (PMC13721193; doi:10.1186/s12913-026-15953-6)
Supplement: Supplementary file 3 [file 12913_2026_15953_MOESM3_ESM.pdf]

Rasch Analyses

The E-Ready (1) consists of 29 items categorized into six dimensions: 1) *conditions for change at the workplace* (8 items), 2) *individual conditions for change* (3 items), 3) *perceived support among the management* (5 items), 4) *readiness among colleagues* (5 items), 5) *perceived consequences on status quo* (5 items), and 6) *workplace attitudes* (3 items). In addition, three single items assess compatibility with current routines, individual commitment for change, and perceived need for change. All dimensions and items included in the E-Ready questionnaire are presented in Supplementary Table 1.

In a Rasch analysis, key aspects of Rasch measurement theory (RMT) are evaluated. This includes the measurement properties *response category functioning, dimensionality, targeting, invariance,* and *reliability* (2, 3). Previous research has shown that the different dimensions of E-Ready are weakly to moderately related (1). Thus, each E-Ready dimension was analyzed separately. Single items were not evaluated with Rasch analyses.

In the following section, each aspect of the RMT is described briefly together with its result. A summary of the Rasch analyses is presented in Supplementary Tables 2 and 3. The sample analyzed in the Rasch analyses consisted of E-Ready responders at the baseline assessment (n=89).

We used RUMM 2030 (version 5.8.1) software to analyze the data according to unrestricted polytomous Rasch models. The significance level was set to <0.05 following Bonferroni corrections (2).

Rasch measurement properties and Rasch analyses results

Response category functioning

We examined the response category thresholds to assess whether the response categories were functioning as intended (i.e., following a sequential order from “not at all” to “a large extent”). Response category thresholds represent the locations on the scale where respondents are equally likely to choose either of the two adjacent categories. Disordered thresholds occur when the locations of two thresholds are not ordered sequentially and vice versa; ordered thresholds occur when the location of two thresholds are in a sequential order. Disordered thresholds can occur due to several reasons, such as if the response categories are not clearly distinct from one another. This may occur, for example, if there are either too many or too few response categories (3). It might also be hard to evaluate whether the response categories work as intended if there are not enough responses in each category. The analyses revealed that all items across all dimensions had ordered thresholds except for item c *(your ability to offer high quality care)* within dimension 5 (*perceived consequences on status quo*).

However, this item was deleted in further analysis due to local dependency issues, so no further adjustments were made.

Dimensionality

Unidimensionality is a basic assumption of RMT indicating that all items for a dimension are measuring the same underlying construct (in our case, the respective E-Ready dimensions). Dimensionality is investigated by the model fit, i.e., the fit of the observed data to the measurement model, and evaluated by different approaches, both statistically and graphically.

Statistical evaluation of dimensionality

Statistically, unidimensionality is assessed by testing how the observed individual item responses match the expected responses from the model. The standardized item fit residual demonstrates the difference between the observed responses and the expected responses from the model. Item fit residual ranges within ±2.5 are considered acceptable (3). Chi-squared statistics can further evaluate the fit to the model. The chi-square focuses on groups of people with the same construct level (so-called class intervals) and examines the discrepancy between their observed values and the expected values from the model. Non-significant chi-square values indicate model fit (2).

For E-Ready, the analyses of each dimension revealed that all the items had an overall mean fit, item fit residuals within acceptable ranges, as well as non-significant chi-square values (after adjustments for local dependencies).

Unidimensionality can also be tested using Smith’s t-test approach. This involves selecting two distinct item sets based on their component loadings from a principal component analysis (PCA) of item fit residuals and estimating the persons’ readiness from each item set. These person measures are then compared using a t-test to evaluate significant differences (4). If less than 5% of the tests are significant, unidimensionality is indicated (5). Regarding E-Ready, dimension 3 (*perceived support among the management*) showed a value of 5.6% while the other dimensions (possible for evaluation with this approach) showed less than 5%.

Furthermore, unidimensionality requires independency of all the items measuring each construct. If two separate items are linked in some way, there is a possibility that an item can predict the answer of another item, which is called local dependency (2). Local dependency is evaluated by examining the fit residual correlations between the items. If two items have correlated fit residuals, it indicates that the answer of one item is relying on another (3). In this study, we used a relative value, as proposed by

Christensen et al (6), as a reference for local dependency. Specifically, residual correlations greater than 0.2 above the average correlation indicated local dependency.

In our data, we identified a cluster of local dependency among items d (*experience among colleges to work with digital solutions*), e (*experience among colleges to implement new work routines*), and f (*adequate competency to implement MINISTOP*), in dimension 1 (*conditions for change at the workplace*). To address this, we tried to merge these items into a "super-item". However, this did not resolve the problem since it affected the functioning of the super-item's response category. Upon closer qualitative examination of these three items, we found that items d and e were generally related to the implementations while all the other items within this dimension specifically addressed the implementation of this particular intervention. Consequently, we decided to delete items d and e. Furthermore, dimension 1 exhibited local dependencies between items a (*sufficient allocated time to implement MINISTOP*) and b (*sufficient staff resources to implement MINISTOP*) as well as between items g (*sufficient support and guidance to adapt MINISTOP to our workplace*) and h (*sufficient training to be able to use and work with MINISTOP*). While items a and b could be merged successfully into a super-item, merging items g and h resulted in disordered thresholds. Given that the residual correlation between items g and h was only slightly above the critical value, we accepted this issue.

For dimension 2 (*individual conditions for change*), we observed a minor local dependency between items b (*competency to work with MINISTOP*) and c (*feeling comfortable to work with MINISTOP*). However, we determined that merging these categories could not be justified qualitatively. Given that the residual correlation was only slightly above the critical value, we accepted this local dependency.

For dimension 3 (*perceived support among management*), we identified a local dependency between items c (*takes an active role in implementing MINISTOP*) and d (*clearly communicates with staff how MINISTOP will be implemented*), a lesser local dependency between items a (*communicates the need for implementing MINISTOP*) and b (*encourages staff to engage in activities to implement MINISTOP*) as well as d (*clearly communicates with staff how MINISTOP will be implemented)* and e (*have insights in how MINISTOP will influence status quo*). Initially, we created a super-item by merging c and d. Following this, we detected one remaining local dependency between items a and b. We attempted to resolve this by creating another super-item, but this resulted in disordered thresholds within the same super-item (a and b). Since the local dependency between items a and b was only slightly above the critical value, we decided to retain the super-item for items c and d and accept the slight local dependency between items a and b.

Dimension 4 (*readiness among colleagues*) also exhibited a local dependency between items d (*work together to adapt current work routines to MINISTOP*) and e (*take collective responsibility for the implementation*). Merging them into a super-item did not resolve this issue since it led to disordered thresholds. After further qualitative examination, we concluded that item d addresses concerns *during* implementation, whereas the others address concerns *before* implementation. Therefore, we removed item d. We also identified a local dependency between item a and item e, but only for nurses with more experience at the current workplace. We accepted this local dependency because the residual correlation was only slightly above the critical value.

Dimension 5 (*perceived consequences on status quo*) exhibited a cluster of local dependencies among items a (*your ability to work in accordance with your values*), b (*your ability to exercise your professional role*), and c (*your ability to offer high quality care*). After further qualitative assessment, we determined that items a and b pertained to the individual role of the nurse while item c pertained more to general care. Based on this, item c was deleted. Since a local dependency still existed between items a and b, these were merged into a super-item. Following this adjustment, there were no further issues with local dependency in dimension 5.

In dimension 6 (*workplace attitudes*), the residual correlations between items a (*among colleagues*) and b (*your attitude*) were slightly above the critical value. We accept this deviation for two reasons: firstly, the exceedance was minimal, and secondly, the dimensions only included three items.

Graphical evaluation of dimensionality

Graphically, unidimensionality was assessed by visually evaluating the item characteristics curves (ICC). The ICC is a graph illustrating how the observed responses deviated from the expected responses at different levels (i.e., class intervals) for the measured variable (i.e., organizational readiness per E-Ready dimension). The closer the observed class interval data is to the theoretical curve, the better the fit to the model (3).

After adjusting for local dependencies, we found the ICC to be acceptable for all the items in dimensions 1 (*conditions for change at the workplace*), 2 (*individual conditions for change*), 3 (*perceived support among the management*), 5 (*perceived consequences on status quo*), and 6 (*workplace attitudes*). However, for item b (*discuss duties that need to be omitted when implementing MINISTOP*) in dimension 4 (*readiness among colleagues*), the persons located on the negative part of the scale tended to respond higher than expected.

In summary, when considering all aspects of dimensionality together, and after adjusting for the local dependencies, we determined that the E-Ready dimensions had an acceptable fit and could be considered unidimensional.

Targeting

Targeting concerns how well the items represent the level of organizational readiness (per E-Ready dimension) reported by the sample and how the person measure distribution covers the levels of organizational readiness. Good targeting implies that good conditions exist for precise measurement (3). Targeting can be visually assessed using person-item maps, wherein good targeting is indicated by the alignment between the distributions of item locations and person measures. Additionally, targeting can be evaluated by comparing the mean person measures with the mean item location (0 logits). Good targeting is inferred when the mean person measure is close to zero. A mean person measure deviating positively indicates that the sample is located at a higher level than the average of the scale, while a lower mean value suggests that the sample reports a lower level than the average of the scale (7).

After adjusting for local dependencies, our data showed that the mean person measure in relation to the mean item location was approximately 0 to 1 logits for dimension 3 (*perceived support among the management*), 4 (*readiness among colleagues*), and 5 (*perceived consequences on status quo*), approximately 2 logits for dimension 1 (*conditions for change at the workplace*) and dimension 2 (*individual conditions for change*), and 3 logits for dimension 6 (*workplace attitudes*).

The visual inspections of person-item maps revealed a ceiling effect for dimensions 2 *(individual conditions for change)* and 6 *(workplace attitudes),* i.e., there were no items representing the persons that reported the highest levels.

Altogether, our sample tended to report a higher level of readiness than what was represented by the items in dimensions 1, 2, and 6. This means that the condition for precise measurements is reduced for parts of the sample.

Invariance

Invariance is a requirement for a good model fit. This means that, irrespective of subgroup of respondents such as gender or age, the items should function in a consistent manner. Invariance is evaluated by “differential item functioning” (DIF). This is a statistical analysis assessed through a two-way ANOVA to determine whether there is a significant difference among mean residuals between the different groups in respondents with similar scores. DIF refers to a situation wherein

different groups (for example different age groups at the same level) respond differently to a particular item (2). In this study, DIF was tested for age (younger = ≤ 44 years vs. older = ≥ 45 years), years of experience at the current workplace (shorter experience = ≤ 5 years vs. longer experience = ≥ 6 years), and years of experience within child healthcare (shorter experience = ≤ 6 years vs. longer experience ≥ 7 years). Gender was not assessed because the responders were all women.

Our data showed that DIF was exhibited for item a (*discuss how work routines need to change when implementing MINISTOP*) in dimension 4 (*readiness among colleagues*) regarding years of experience at the current child healthcare center. Nurses with more experience answered higher than expected, while nurses with less experience answered lower than expected. To address this, we conducted an item split, meaning that the item was measured differently for the different groups. Afterwards, there were no longer any issues with DIF, and all the E-Ready dimensions are considered to work invariantly.

Reliability

Reliability was assessed as the Person Separation Index (PSI). The PSI indicates how good the test can separate persons based on its person measures. The PSI can be interpreted equally to Cronbach alpha, with ranges between 0-1, where a value close to 1 indicates a greater ability to detect differences between persons. A value of 0.70 is considered acceptable for group use and 0.85 for individual use (7).

In our data, the PSI for the different E-Ready dimensions ranged from 0.59 to 0.90.
The low PSI values for dimension 2 (0.68) and dimension 6 (0.59) could be attributed primarily to the fact that these dimensions comprised only three items; with so few items, limited information was available when estimating person measures.

Additional file 3
Rasch analyses

Supplementary Table 1. Dimensions and items included in the E-Ready questionnaire.

E-Ready dimension	Items included in the dimensions
**1. Conditions for change at the workplace**	How do you perceive the resources in your workplace to implement MINISTOP?<br>a) Sufficient allocated time to implement MINISTOP<br>b) Sufficient staff resources to implement MINISTOP<br>c) Clear goals on how to implement MINISTOP<br>d) Experience among colleges to work with digital solutions<br>e) Experience among colleges to implement new work routines<br>f) Adequate competency to implement MINISTOP<br>g) Sufficient support and guidance to adapt MINISTOP to our workplace<br>h) Sufficient training to be able to use and work with MINISTOP
**2. Individual conditions for change**	How do you perceive your own resources to use MINISTOP in your work?<br>a) Experience to work with digital solutions<br>b) Competency to work with MINISTOP<br>c) Feeling comfortable to work with MINISTOP
**3. Perceived support among the management**	To what extent do you perceive your manager…<br>a) Communicates the need for implementing MINISTOP<br>b) Encourages staff to engage in activities to implement MINISTOP<br>c) Takes an active role in implementing MINISTOP<br>d) Clearly communicates with staff how MINISTOP will be implemented<br>e) Have insights in how MINISTOP will influence status quo
**4. Readiness among colleagues**	To what extent do you and your colleague…<br>a) Discuss how work routines need to change when implementing MINISTOP<br>b) Discuss duties that need to be omitted when implementing MINISTOP<br>c) Discuss new duties that need to be done when implementing MINISTOP<br>d) Work together to adapt current work routines to MINISTOP<br>e) Take collective responsibility for the implementation
**5. Perceived consequences on status quo**	In what way do you think implementing MINISTOP will impact your daily routines regarding…<br>a) Your ability to work in accordance with your values<br>b) Your ability to exercise your professional role<br>c) Your ability to offer high quality care<br>d) Your ability to manage all your work<br>e) How you think MINISTOP aligns with the current workflow at your workplace?
**6. Workplace attitudes**	How do you perceive attitudes to implement MINISTOP at your workplace?<br>a) Among colleagues<br>b) Your attitude<br>c) Your manager's attitude
**Singe items**	1) How well does MINISTOP fit with current work routines?<br>2) What role would you like to have when MINISTOP is being implemented at your workplace?<br>3) How do you perceive the value of implementing MINISTOP at your workplace?

Additional file 3
Rasch analyses

Supplementary table 2. Results from Rasch analyses per dimension of E-Ready (1), n=89.

	Rasch component	Fit criteria	Dimension 1, conditions for change at the workplace. Initial run. 8 items.	Dimension 1, conditions for change at the workplace. Final run. 5 items (deleted items d and e, super item with items a and b.	Dimension 2, individual conditions for change. 3 items. Initial and final run.	Dimension 3, perceived support among the management. Initial run. 5 items.	Dimension 3, perceived support among the management, Final run. 4 items (super item with items c and d).
**Unidimensionality**	**Individual item fit.** The degree to which the observed individual item responses matched the expected responses from the Rasch model.	Fit residual ± 2.5 (3). Nonsignificant chi-square probability values, Bonferroni-adjusted (2).	Item d Fit Residual 2.7. All others within ± 2.5. Nonsignificant chi-square probability values, Bonferroni-adjusted.	All items Fit Residuals within ± 2.5. Nonsignificant chi-square probability values, Bonferroni-adjusted.	All items Fit Residuals within ± 2.5. Nonsignificant chi-square probability values, Bonferroni-adjusted.	All items Fit Residuals within ± 2.5. Nonsignificant chi-square probability values, Bonferroni-adjusted.	All items Fit Residuals within ± 2.5. Nonsignificant chi-square probability values, Bonferroni-adjusted.
	**Smith t-test.** Tests whether the person measures, which are based on item sets from a PCA of residuals, differ from each other.	≤ 5% of the t-tests significant (5).	12.4%	1.1%	1.2%	5.6%	5.6%
	**Item characteristic curves** The degree of alignment between observed and expected item responses at various levels of organizational readiness.	Visual check of item characteristic curves. The observed values should closely follow the theoretical curve (2).	Item d: tendency of a person at the more negative part of the scale to answer higher than the expected value. Persons on the more positive part of the scale respond lower than expected.	The observed values fit relatively closely to the theoretical curve.	The observed values fit relatively closely to the theoretical curve.	The observed values fit relatively closely to the theoretical curve.	The observed values fit relatively closely to the theoretical curve.
	**Overall fit of items.** The degree to which the observed individual item responses matched the expected responses from the Rasch model.	Perfect fit = mean of 0 and SD of 1 (2).	Mean -0.02, SD 1.4.	Mean 0.2, SD 1.4.	Mean 0.09, SD 1.4.	Mean -0.15, SD 1.3.	Mean 0.05, SD 1.1.
	**Local dependency.** Whether item responses exhibit independence from one another.	Residual correlations greater than 0.20 above the average correlations (6).	Local dependency items a and b, items d and e, items d and f, items e and f, items g and h.	Items g and h have a residual correlation slightly over the critical value (0.07, critical value -0.01)	Items b and c have a residual correlation slightly over the critical value (-0.24, critical value -0.28).	Local dependency items c and d. Items a and b have a residual correlation slightly over the critical value (0.03 critical value -0.02)	Items a and b have a residual correlation slightly over the critical value (0.06, critical value -0.09),

Additional file 3
Rasch analyses

						Residual correlation between items d and e 0.02.	
**Response categories**	**Thresholds.** Whether the response categories and thresholds work as intended.	Ordered thresholds (2).	Ordered thresholds.	Ordered thresholds.	Ordered thresholds.	Ordered thresholds.	Ordered thresholds.
**Targeting**	**Targeting.** To what extent the items in a scale represent the levels of the construct as reported by the sample.	Mean person measure should be close to mean item location (7).	Mean person measure 2.0. At the negative end of the scales, there are no persons located at the item thresholds. At the positive end, there are no item thresholds at the person measures.	Mean person measure 2.2. At the negative end of the scales, there are no persons located at the item thresholds. At the positive end, there are no item thresholds at the person measures.	Mean person measure 2.0. At the negative end of the scales, there are few persons located at the item thresholds. At the very positive end, there are no item thresholds at the person measures.	Mean person measure 0.8. Relatively well targeted; however, there are no item thresholds at person measures at the very positive or negative ends of scale.	Mean person measure -0.7. Relatively well targeted; however, there are no item thresholds at person measures at the very positive or negative ends of scale.
**Invariance**	**Differential item functioning (DIF).** Whether the items work in a similar way in different subgroups of respondents	Nonsignificant probability value, Bonferroni-adjusted (2).	No DIF	No DIF	No DIF	No DIF	No DIF
**Reliability**	**Person separation index.** The capability to differentiate among persons with different levels of organizational readiness.	0.70 acceptable for group use and 0.85 for individual use (7).	0.80	0.83	0.68	0.90	0.90

Additional file 3
Rasch analyses

Supplementary table 3. Continuing results from Rasch analyses per dimension of E-Ready (1), n=89.

	Rasch component	Fit criteria	Dimension 4, readiness among colleagues. Initial run. 5 items.	Dimension 4, readiness among colleagues. Final run. 4 items (deleted item d, item split item a (experience at current workplace).	Dimension 5, perceived consequences on status quo. Initial run. 5 items.	Dimension 5, perceived consequences on status quo. Final run. 3 items (deleted item c, super-item a/b).	Dimension 6, workplace attitudes. Initial and final run. 3 items.
**Unidimensionality**	**Individual item fit.** The degree to which the observed individual item responses matched the expected responses from the Rasch model.	Fit residual ± 2.5 (3). Nonsignificant chi-square probability values, Bonferroni-adjusted (2).	All items Fit residuals within ± 2.5. Nonsignificant chi-square probability values, Bonferroni-adjusted.	All items Fit residuals within ± 2.5. Nonsignificant chi-square probability values with Bonferroni-adjustments	All items Fit residuals within ± 2.5. Nonsignificant chi-square probability values, Bonferroni-adjusted.	All items Fit residuals within ± 2.5. Nonsignificant chi-square probability values, Bonferroni-adjusted.	All items Fit residuals within ± 2.5. Nonsignificant chi-square probability values, Bonferroni-adjusted.
	**Smith t-test.** Tests whether the person measures, which are based on item sets from a PCA of residuals, differ from each other.	≤ 5% of tests significant (5).	3.4%	N/A	6.8%	2.3%	2.3%
	**Item characteristic curves** The degree of alignment between observed and expected item responses at various levels of organizational readiness.	Visual check of item characteristic curves. The observed values should closely follow the theoretical curve (2).	Item b: persons on the negative part of the scale answer higher than expected.	Item b: persons on the negative part of the scale answer higher than expected.	The observed values fit relatively closely to the theoretical curve.	The observed values fit relatively closely to the theoretical curve.	The observed values fit relatively closely to the theoretical curve.
	**Overall fit of items.** The degree to which the observed individual item responses matched the expected responses from the Rasch model.	Perfect fit = mean of 0 and SD of 1 (2).	Mean -0.06, SD 0.8.	Mean 0.4, SD 0.6.	Mean -0.03, SD 1.3.	Mean 0.2, SD 1.6	Mean -0.7, SD 1.3.
	**Local dependency.** Whether item responses exhibit independence from another.	Residual correlations greater than 0.20 above the average correlations (6).	Local dependency items d and e.	Residual correlation among item e and item a slightly over the critical value for nurses with more experience at current workplace (-0.04, critical value -0.10).	Local dependency items b and c. Items a and b have a residual correlation slightly over the critical value (0.058, critical value -0.039).	No local dependency.	Residual correlation among items a and b slightly over the critical value (-0.18, critical value -0.28).

Additional file 3
Rasch analyses

**Response categories**	**Thresholds.** Whether the response categories and thresholds work as intended.	Ordered thresholds (2).	Ordered thresholds.	Ordered thresholds.	Disordered thresholds item c: Category 1 never highest probability.	Ordered thresholds.	Ordered thresholds.
**Targeting**	**Targeting.** To what extent the items in a scale represent the levels of the construct as reported by the sample.	Mean person measure should be close to mean item location (7).	Mean person measure -0.02. Relatively well targeted; however, there are no item thresholds at person measures at very positive or negative ends of the scale.	Mean person measure -0.24. Relatively well targeted; however, there are no item thresholds at person measures at the very positive or negative ends of scale.	Mean person measure 1.94. At the negative end of the scale, there are no persons located at the item thresholds. At the positive end, there are few item thresholds at the person measures.	Mean person measure 1.00. At the negative end of the scale, there are no persons located at the item thresholds. At the positive end, there are few item thresholds at the person measures.	Mean person measure 3.0. At the negative end of the scales there are no persons located at the item thresholds. At the positive end there are no item thresholds at the person measures.
**Invariance**	**Differential item functioning (DIF).** Whether the items work in a similar way in different subgroups of respondents.	Nonsignificant probability value, Bonferroni-adjusted (2).	Item a DIF regarding years of experience at current workplace. Nurses with more experience within the workplace answer higher than expected. Nurses with less experience answer lower than expected.	No DIF	No DIF	No DIF	No DIF
**Reliability**	**Person separation index.** The capability to differentiate among persons with different levels of organizational readiness.	0.70 acceptable for group use and 0.85 for individual use (7).	0.89	0.86	0.78	0.73	0.59

Additional file 3
Rasch analyses

References

1. Dannapfel P, Thomas K, Chakhunashvili A, Melin J, Lagerros YT. A Self-help tool to facilitate implementation of eHealth initiatives in health care (E-Ready): Formative evaluation. JMIR Form Res. 2022;6(1):e17568.
2. Andrich D, Marais I. A course in Rasch measurement theory. Measuring in the educational, social and health sciences. Singapore: Springer; 2019.
3. Hobart J, Cano S. Improving the evaluation of therapeutic interventions in multiple sclerosis: The role of new psychometric methods. Health Technol Assess. 2009;13:1-177.
4. Tennant A, Küçükdeveci AA. Application of the Rasch measurement model in rehabilitation research and practice: Early developments, current practice, and future challenges. Front Rehabil Sci. 2023;4:1208670.
5. Smith Jr EV. Detecting and evaluating the impact of multidimensionality using item fit statistics and principal component analysis of residuals. J Appl Meas. 2002;3(2):205-31.
6. Christensen KB, Makransky G, Horton M. Critical values for Yen's Q(3): Identification of local dependence in the Rasch model using residual correlations. Appl Psychol Meas. 2017;41(3):178-94.
7. Tennant A, Conaghan PG. The Rasch measurement model in rheumatology: What is it and why use it? When should it be applied, and what should one look for in a Rasch paper? Arthritis Rheum. 2007;57(8):1358-62.
